# Polycyclic aromatic hydrocarbons: bioaccumulation in dragonfly nymphs (Anisoptera), and determination of alkylated forms in sediment for an improved environmental assessment

**DOI:** 10.1038/s41598-020-67355-1

**Published:** 2020-07-02

**Authors:** Viviane Girardin, Merete Grung, Sondre Meland

**Affiliations:** 1 Norwegian Institute for Water Research (NIVA), Gaustadalléen 21, 0349 Oslo, Norway; 2 Faculty of Environmental Sciences and Natural Resource Management, Norwegian University for Life Sciences (NMBU), PO 5003, 1432 Ås, Norway

**Keywords:** Environmental impact, Environmental sciences

## Abstract

Road runoff carries a mixture of contaminants that threatens the quality of natural water bodies and the health of aquatic organisms. The use of sedimentation ponds is a nature-based solution for the treatment of road runoff. This study assessed the concentration of polycyclic aromatic hydrocarbons (PAHs) and their alkylated homologues in sediment from seven highway sedimentation ponds and three natural urban ponds. In addition, the study explored the bioaccumulation of PAHs in dragonfly nymphs (Anisoptera). Finally, biota-sediment accumulation factors (BSAFs) were estimated. The results revealed a significant difference in the concentrations of 16 priority PAHs in sediment, with overall higher levels in sedimentation ponds (2,911 µg/kg on average) compared to natural urban ponds (606 µg/kg on average). PAH levels increased substantially once alkylated homologues were considered, with alkylated comprising between 42 and 87% of the total PAH in sediment samples. These results demonstrate the importance of alkylated forms in the environmental assessment of PAHs. The bioaccumulation assessment indicates that dragonfly nymphs bioaccumulate PAHs to a certain degree. It is not clear, however, whether they metabolize PAHs. BSAF results ranged from approx. 0.006 to 10 and indicate that BSAFs can be a powerful tool to determine the functionality of sedimentation ponds.

## Introduction

During traffic-related activities, a complex mixture of inorganic and organic contaminants is released into the environment. Some of these contaminants remain in the air or settle on the ground, being eventually washed off by rain^1,2^. Road runoff and tunnel wash water, enriched with contaminants, can eventually reach natural water bodies, threatening the water quality, and the health of all organisms dependent directly or indirectly on these systems.

Among many traffic-related contaminants, polycyclic aromatic hydrocarbons (PAHs) are probably the most studied group of organic contaminants. Tire and asphalt wear, and incomplete combustion of fuel are the main sources of PAHs from traffic^3^. PAHs are found in complex mixtures, with those of pyrogenic origin (from incomplete combustion) being mainly parent PAHs (without alkyl groups, heteroatoms or hydroxides), whereas those of petrogenic origin (from petroleum derivate) are associated with high levels of alkylated forms^4^.

Several PAHs and their metabolites are known to be potentially carcinogenic and mutagenic^5–7^, and some exhibit photo-induced toxicity^8,9^. Consequently, several PAHs have been added to the list of substances of concern in environmental risk assessment and monitoring. A list of 16 parent PAHs issued by the U.S. Environmental Protection Agency (EPA) in the 1970s, and referred in this study as PAH-16, is often used as a standard set of PAHs for environmental analysis^10^. A more recent study has, however, recommended a list of 40 PAHs when considering toxicity in the environment, including several alkylated homologues^11^. Some alkylated PAHs are reported to be more toxic than their parental forms^12, 13^.

A variety of treatment solutions can be established to reduce the impacts of PAHs and other traffic related contaminants present in road runoff on water bodies. One of the most commonly used mitigation methods is the use of nature-based sedimentation ponds. These low-cost, sustainable urban drainage systems are built to retain particle-bound contaminants, improving the quality of the runoff before its discharge into natural water bodies^14^. Quite rapidly, these ponds are colonized by a wide range of organisms, leading to the formation of small complex ecosystems in rather urban zones^15,16^. These systems’ apparent ability to support and enhance biodiversity is often acknowledged as a positive ecosystem service by, e.g. road and urban planners. However, some have argued that biota inhabiting such nature-based sedimentation ponds can be harmed by the contaminants they are inevitably exposed to in these systems^17,18^. For example, our research group recently documented increased genotoxicity in nymphs of dragonflies (Odonata: Anisoptera, *Aeshna *sp.) living in sedimentation ponds compared to those living in natural ponds^19^. Whereas vertebrates metabolize PAHs^20–23^, metabolism efficiency varies among invertebrates^24–26^. Furthermore, PAHs may potentially be transported out of the ponds by organisms with both aquatic and terrestrial life stages. Dragonflies have an aquatic life stage that can last years, followed by a short, terrestrial, adult life stage. They are, therefore, potential vectors of bioaccumulative contaminants from the aquatic to terrestrial environments.

In the present study, we determined the concentration of PAHs and their alkylated homologues in sediment, and assessed bioaccumulation and biotransformation of PAHs in dragonfly nymphs from the genus *Aeshna*. *Aeshna* nymphs spend most of their time hidden between the aquatic vegetation where they prey on various invertebrates and small vertebrates such as tadpoles. Sediment and dragonfly nymphs from seven nature-based sedimentation ponds and three natural urban ponds were included in the study.

## Results

### Levels of PAHs and their estimated alkylated homologues in sediment

**Figure 1:**
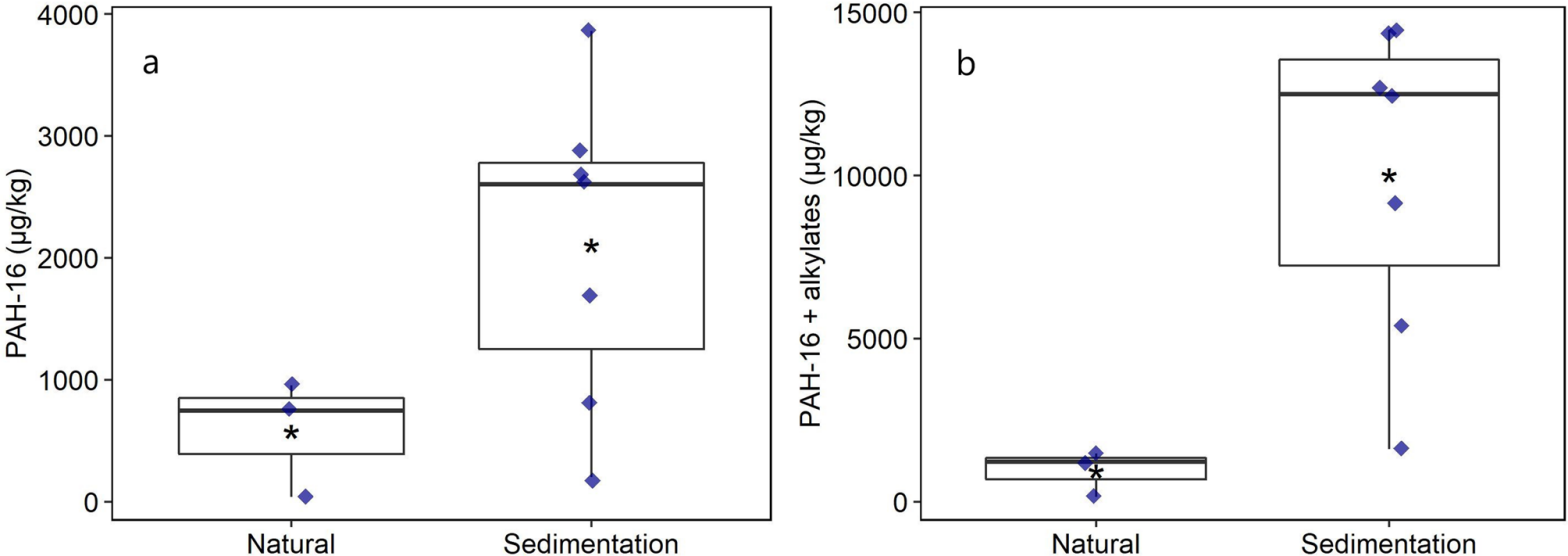
Concentrations of PAH-16 (**a**), and PAH-16 and alkylated PAHs (**b**) in sediment samples from sedimentation and natural urban ponds. Concentration in µg/g (dry weight). Black horizontal line represents the median. Asterisk represents the mean. Each data point represents a pond. Natural, n = 3. Sedimentation, n = 7. PAHs below the detection limit were set to  ½ the detection limit value.

The levels of individual parental and estimated alkylated PAHs are shown in Table 1. Ponds were grouped as SED—*n* for sedimentation ponds and NAT—*n* for natural urban ponds. All sites are described in Table 2. Analysis of the concentrations of PAHs in sediment samples showed overall higher concentrations of PAH-16 in sedimentation compared to natural urban ponds (Fig. 1a—Welch’s Two Sample t-test, p = 0.025). The exceptions were the sedimentation ponds SED—5 and 7, in which PAH levels were lower than in some of the natural urban ponds. The highest levels of PAHs were detected in the samples from SED—1, 2, 4, and 6. Inclusion of alkylated PAHs led to a substantial increase in total PAH levels (Fig. 1b—Welch’s Two Sample t-test, p = 0.002). All sedimentation ponds had higher concentrations of total PAHs compared to natural urban ponds once alkylated forms were included.

**Figure 2:**
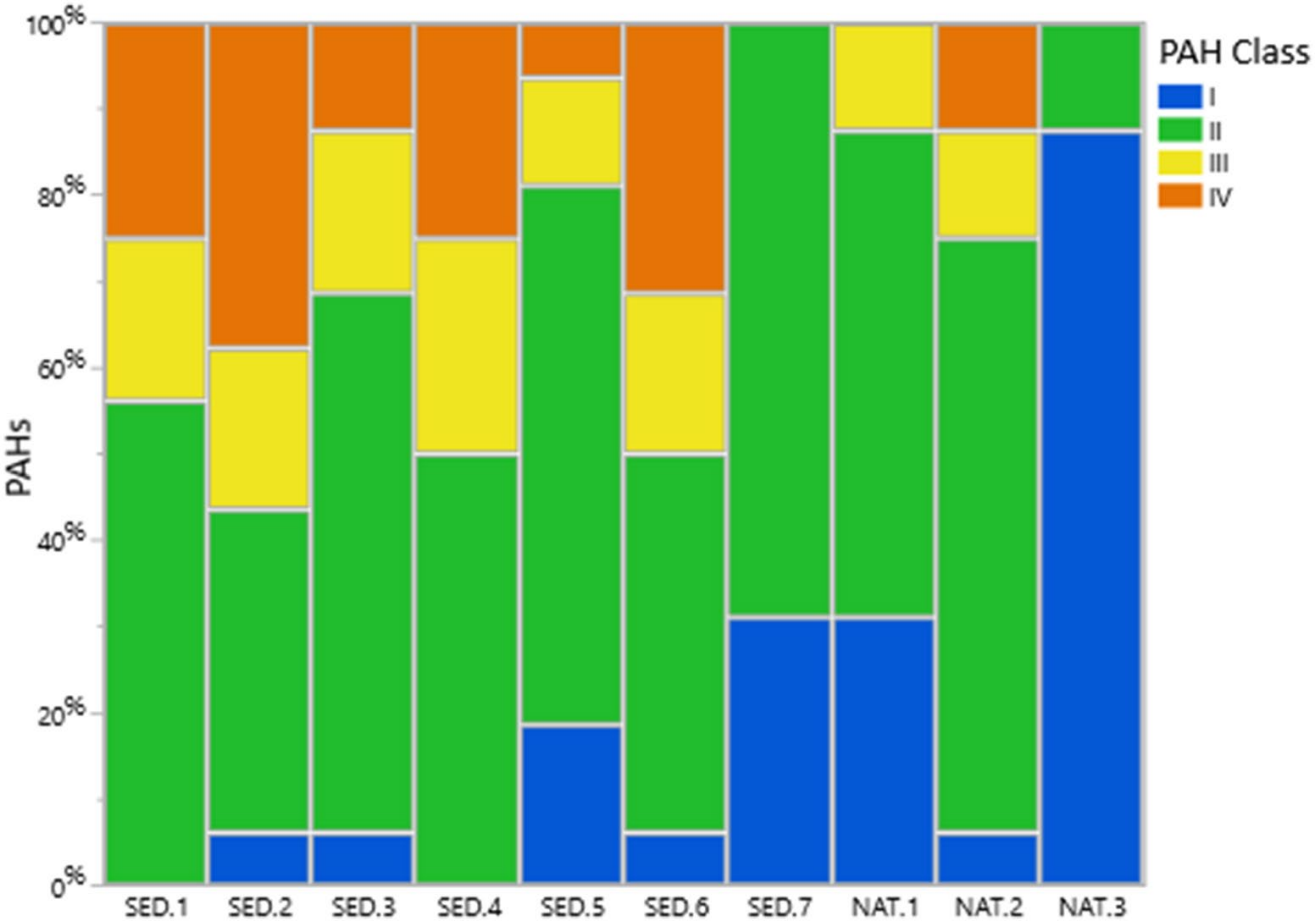
Bar plot of the classification of PAH-16 per sediment sample (n-16) according to the EQS set by the Norwegian Committee of Directorates for the Water Framework Directive^27^.

**Figure 3:**
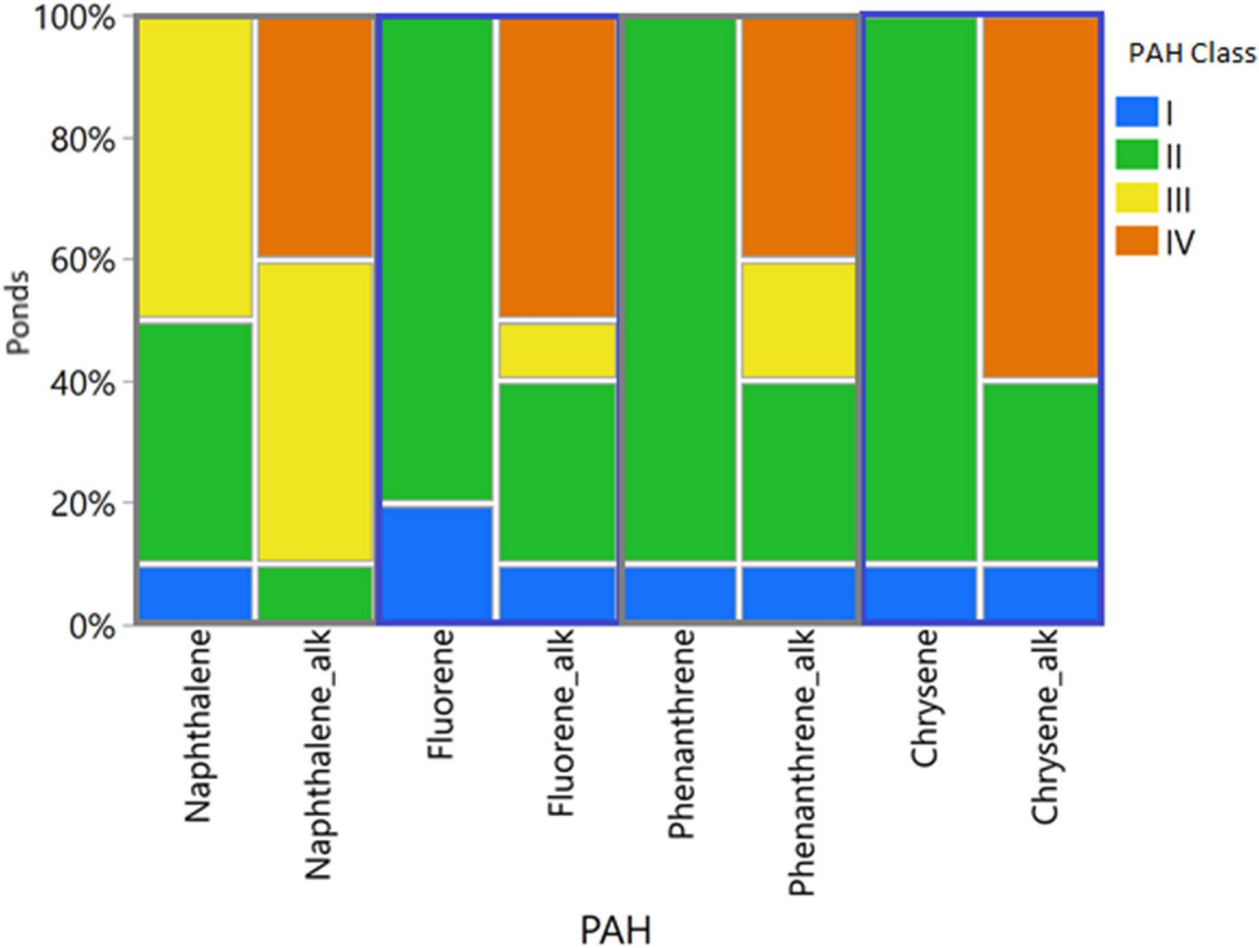
Bar plot of the classification of PAHs phenanthrene, naphthalene, fluorene, chrysene, and their alkylated PAHs per pond, excluding NAT-3 (n = 9). EQS set by the Norwegian Committee of Directorates for the Water Framework Directive^27^.

PAH-16 levels in the sediments were classified according to the environmental quality standards (EQSs) set by the Norwegian Committee of Directorates for the Water Framework Directive^27^. The classification ranges from I to V, according to the PAH concentrations. Exposure to PAH concentrations classified as levels I or II are considered low, and no toxic effects are expected. Exposure to levels III and IV might cause chronic and acute effects in organisms, respectively ^28^. Many of the PAHs detected in our sediment samples were classified as III and IV (Fig. 2). Inclusion of alkylated results in the concentrations of phenanthrene, naphthalene, fluorene, and chrysene caused these PAHs to change their classification from class II to class IV in several cases (Fig. 3).

**Table 1:** Concentration of PAHs detected in sediment samples; “i” means interference during analysis and result is not available.

**PAHs (µg/kg dry weight)**	**Ponds**									
	**SED—1**	**SED—2**	**SED—3**	**SED—4**	**SED—5**	**SED—6**	**SED—7**	**NAT—1**	**NAT—2**	**NAT—3**
**Naphthalene Class**	**III**	**III**	**III**	**III**	**II**	**III**	**II**	**II**	**II**	**II**
**Naphthalene**^**1,3**^	**69**	**84**	**53**	**54**	** < 15**	**47**	** < 10**	** < 20**	** < 10**	** < 10**
**Fluorene Class**	**III**	**III**	**III**	**III**	**II**	**III**	**II**	**II**	**II**	**II**
**Fluorene**^**1,4**^	**83**	**67**	**41**	**32**	**18**	**64**	** < 7**	**29**	**23**	** < 1**
**Phenanthrene Class**	**II**	**II**	**II**	**II**	**II**	**II**	**II**	**II**	**II**	**I**
**Phenanthrene**^**1,4**^	**270**	**512**	**213**	**185**	**69**	**283**	**13**	**95**	**80**	**2.8**
**Chrysene Class**	**II**	**II**	**II**	**II**	**II**	**II**	**II**	**II**	**II**	**I**
**Chrysene**^**1,2,4**^	**215**	**215**	**73**	**176**	**62**	**118**	**17**	**22**	**79**	**3**
**Fluoranthene Class**	**II**	**IV**	**II**	**II**	**II**	**IV**	**II**	**II**	**II**	**I**
**Fluoranthene**^**1,3**^	**392**	**622**	**221**	**386**	**107**	**415**	**22**	**333**	**161**	**5.3**
**Pyrene Class**	**III**	**IV**	**III**	**III**	**III**	**III**	**II**	**III**	**III**	**I**
**Pyrene**^**1,4**^	**659**	**1,190**	**468**	**641**	**214**	**825**	**30**	**130**	**108**	**4**
**Benzo(a)anthracene Class**	**III**	**III**	**II**	**III**	**II**	**III**	**II**	**II**	**II**	**I**
**Benzo(a)anthracene**^**1,2,4**^	**117**	**98**	**39**	**113**	**21**	**67**	**6.8**	**13**	**50**	**2**
**Benzo(b,j)fluoranthene Class**	**IV**	**IV**	**IV**	**IV**	**I**	**IV**	**I**	**I**	**IV**	**I**
**Benzo(b,j)fluoranthene**^**1,2,3**^	**351**	**281**	**142**	**306**	**88**	**221**	**30**	**42**	**166**	**5.5**
**Benzo(k)fluoranthene Class**	**II**	**I**	**I**	**II**	**I**	**I**	**I**	**I**	**I**	**I**
**Benzo(k)fluoranthene**^**c, 1,2,3**^	**95**	**67**	**33**	**97**	**18**	**54**	**7.7**	**7.0**	**50**	**2**
**Benzo(a)pyrene Class**	**II**	**II**	**II**	**II**	**II**	**II**	**II**	**II**	**II**	**I**
**Benzo(a)pyrene**^**1,2,3**^	**149**	**128**	**73**	**164**	**40**	**108**	**13**	**17**	**61**	**2.4**
**Indeno(1,2,3-cd)pyrene**	**IV**	**IV**	**II**	**IV**	**II**	**IV**	**I**	**I**	**IV**	**I**
**Indeno(1,2,3-cd)pyrene**^**1,2,3**^	**133**	**117**	**54**	**134**	**35**	**78**	**13**	** < 20**	**80**	**2.6**
**Dibenz(ac/ah)anthracene Class**	**III**	**III**	**III**	**III**	**II**	**III**	**I**	**I**	**II**	**I**
**Dibenz(ac/ah)anthracene**^**1,2,4**^	** < 50**	** < 50**	** < 30**	**44**	** < 15**	** < 40**	** < 10**	** < 8**	**17**	** < 1**
**Benzo(ghi)perylene Class**	**IV**	**IV**	**IV**	**IV**	**IV**	**IV**	**II**	**I**	**II**	**I**
**Benzo(ghi)perylene**^**2,3**^	**230**	**342**	**211**	**262**	**117**	**235**	**24**	** < 20**	**44**	** < 1**
**Anthracene Class**	**IV**	**IV**	**III**	**IV**	**III**	**IV**	**II**	**III**	**III**	**I**
**Anthracene**^**2,3**^	**51**	**47**	**16**	**40**	**12**	**41**	**2.2**	**11**	**19**	** < 1**
**Acenaphthylene Class**	**II**	**III**	**II**	**II**	**II**	**II**	**II**	**II**	**II**	**II**
**Acenaphthylene**^**2,4**^	**26**	**36**	**12**	**22**	** < 15**	** < 15**	** < 10**	** < 20**	** < 10**	** < 5**
**Acenaphthene Class**	**II**	**II**	**II**	**II**	**II**	**II**	**II**	**II**	**II**	**II**
**Acenaphthene**^**2,4**^	**21**	**29**	** < 15**	** < 25**	** < 15**	** < 40**	** < 6**	** < 10**	**6.8**	** < 6**
Dibenzothiophene	20	33	17	19	< 4	19	< 2	33	6.3	< 1
Benzo[e]pyrene	368	406	213	331	125	345	40	20	72	2.6
Perylene	54	65	< 40	80	23	74	11	NA	15	< 5
*∑PAH*	*3,353*	*4,389*	*1964*	*3,111*	*1,013*	*3,089*	*275*	*850*	*1,058*	*63*
*∑PAH16*	***2,911***	***3,885***	***1694***	***2,681***	***861***	***2,651***	***222***	***797***	***965***	***55***
C1—Naphthalene	100	87	64	51	19	45	13	< 20	< 10	< 10
C2—Naphthalene	581	295	368	284	386	287	207	106	39	< 60
C3—Naphthalene	2,830	1,340	2,520	905	1,660	1,010	761	< 100	57	< 100
C4—Naphthalene	810	428	1,020	248	332	353	113	< 70	< 10	i
C1—Fluorene	269	211	231	109	50	184	< 10	< 20	21	i
C2—Fluorene	470	454	624	268	90	478	< 15	< 30	27	i
C3—Fluorene	802	< 1,100	1,410	511	< 190	< 980	< 40	< 160	< 60	i
C1—Phenanthrene	366	480	428	219	67	425	< 11	46	40	< 5
C2—Phenanthrene	1,370	1900	1,610	213	356	1,570	< 40	< 60	113	< 5
C3—Phenanthrene	902	1,030	839	674	234	1,050	i	< 60	65	< 5
C4—Phenanthrene	335	250	< 300	490	< 140	390	< 20	< 30	17.5	i
C1—Chrysene	858	1,090	521	749	309	993	83	< 40	80	i
C2—Chrysene	1,780	2,490	1,280	1,740	857	2,610	171	< 50	< 50	i
C1—Dibenzothiophene	119	120	88	67	16	101	< 6	18	12	< 10
C2—Dibenzothiophene	718	785	501	493	142	690	< 20	32	55	< 10
C3—Dibenzothiophene	1,340	1,590	996	886	372	1,550	< 60	< 60	103	< 10
*∑Alkylated PAHs*	*13,670*	*13,683*	*12,817*	*7,926*	*5,224*	*12,735*	*1572*	*935*	*766*	*i*
*∑PAHs* + *alkylated PAHs*	*17,023*	*18,072*	*14,781*	*11,037*	*6,237*	*15,824*	*1847*	*1785*	*1824*	*-*

Classification according to Norwegian Committee of Directorates for the Water Framework Directive^27^. PAH-16 are given in bold. *SED* sedimentation pond, *NAT * natural pond. Specific priority substances are marked as:1—U.S. EPA 16; 2—IARC classification; 3—European priority pollutant as defined by the European Commission; 4—Norwegian river basin specific pollutants. Sources:^27,29–35^.

**Table 2:** General information of the various ponds. Geographic coordinates obtained from http://www.norgeskart.no (Access 2019.06.01). Traffic density measured as annual average daily traffic (AADT), in units of cars per day, and was obtained from www.vegkart.no (access 2019.06.05).

**Code**	**Pond**	**Coordinates**	**AADT (unit/day)**	**Size (m**^**2**^**)**	**Terrestrial vegetation**	**Aquatic vegetation**
SED—1	Fornebu	59.90115, 10.62591	23,800	Forebay 145; Main 480	Very dense vegetation around the whole pond	Moderate
SED—2	Taraldrud North	59.80933, 10.84031	50,344	780	Dense around the whole pond	Moderate
SED—3	Taraldrud Junction	59.79662, 10.84075	50,339	1,400	Dense around the whole pond	Moderate
SED—4	Taraldrud South	59.78405, 10.84002	50,339	474	Dense around the whole pond	Moderate
SED—5	Nøstvedt	59.77153, 10.83268	46,915	Forebay 40; Main 340	Dense around the main pond, light around forebay	Moderate
SED—6	Vassum	59.70988, 10.73669	66326^1^	Forebay 68; Main 363	Very dense vegetation around the main pond	Moderate
SED—7	Tenor	59.57755, 11.26207	12,000	Forebay 175; Main 480	Very dense around the whole pond	Dense
NAT—1	Båntjern	59.96119, 10.69742	NA	500	Dense around the whole pond	Moderate
NAT—2	Møllesvingen	59.94762, 10.73823	NA	320	Dense around the whole pond	Dense
NAT—3	Svarta	59.88792, 10.79212	NA	1,200	Dense around the whole pond	Dense

^1^Based on the sum of AADT of the three tunnels Nordbytunnelen (E6), Smiehagentunnelen (E6) and Vassumtunnelen (E134).

### PAHs in dragonfly nymphs

#### Concentrations

Concentrations of parent PAHs in tissues, exuvia, and whole dragonfly nymphs were determined in order to investigate bioaccumulation (Supplementary table S1). PAHs acenaphthene, acenaphthylene, phenanthrene, fluoranthene, and pyrene were detected in at least 80% of all samples, and for that reason they were the only ones used in the following statistical analyses. *Early instars* were not found in NAT—2, and thus not included.

**Figure 4:**
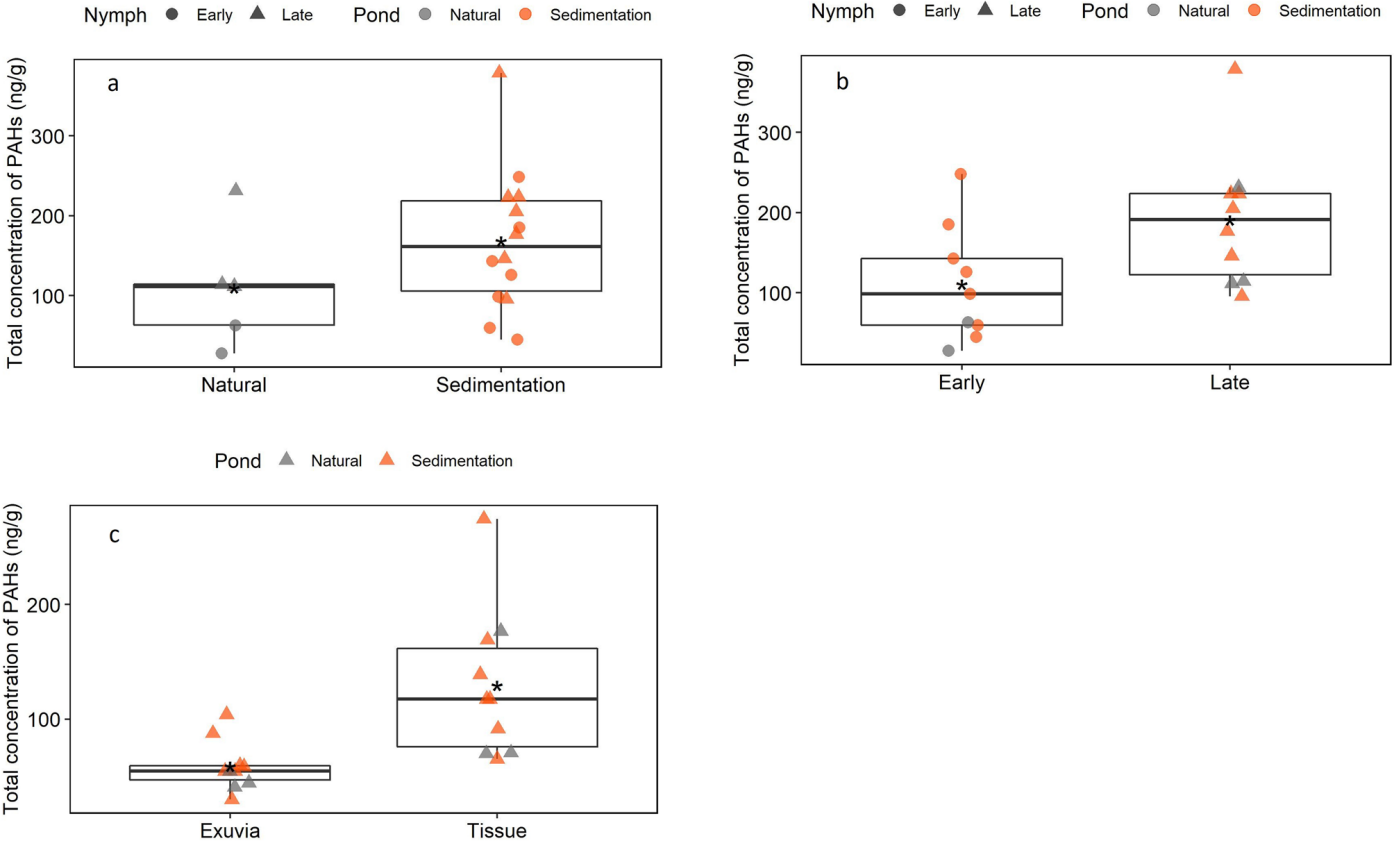
Boxplots of the sum of the concentration of PAHs in nymphs from sedimentation and natural urban ponds (**a**), early and late instars (**b**), and exuvia and tissues of late instars (**c**). Concentration in ng/g (dry weight). Black horizontal line represents the median. Asterisk represents the mean. Late, n = 10, Early, n = 9. Exuvia: n = 10, Tissue; n = 1.

A comparison between the concentrations of PAHs in nymphs from sedimentation and natural urban ponds did not reveal a significant difference between groups (Fig. 4a*—*Welch’s Two Sample t-test, p-value = 0.2). The result was caused by particularly high concentrations of phenanthrene, fluoranthene, and pyrene in some of the *Late instars* from NAT—1.

A comparison between PAH concentrations in early instars and late instars nymphs, independent of location, showed significantly higher levels in late instar nymphs in comparison to early instars (Fig. 4b—Welch’s Two Sample t-test, p = 0.04). There was also a significant difference between exuvia and tissues sampled from the same nymphs, with higher levels of PAHs in the tissues (Fig. 4c—Welch’s Two Sample t-test, p = 0.007).

#### PAH metabolites

**Figure 5:**
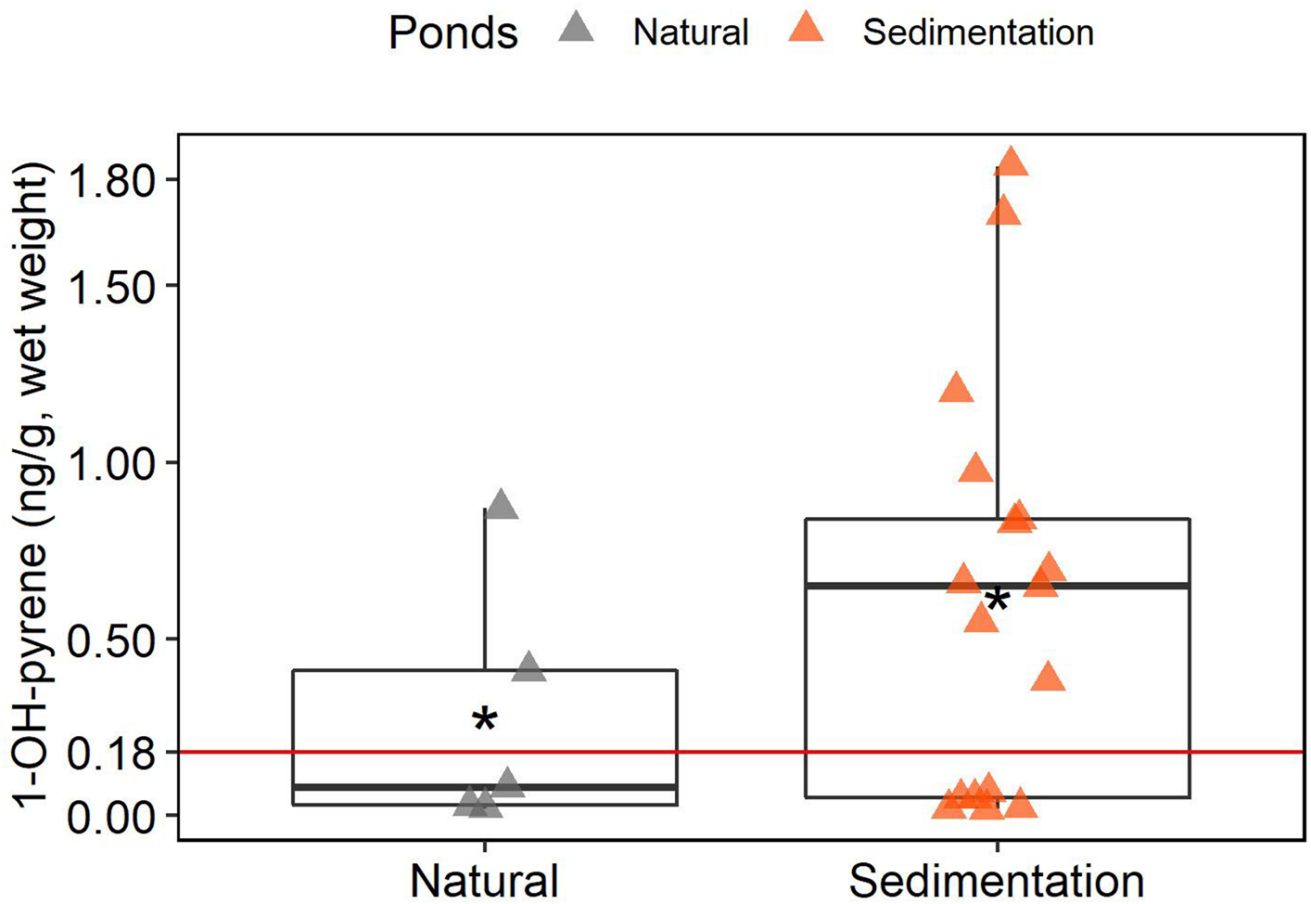
Box plot of the levels of 1-OH-pyrene detected in dragonfly nymphs. Black horizontal line represents the median. Asterisk represents the mean. Natural: n = 5, Sedimentation; n = 17. Red line defines the limit of detection. Results below LOD were set to ½ its value.

Levels of 1-OH-phenanthrene, 1-OH-pyrene and 3-OH-benzo[a]pyrene were analysed in nymph haemolymph. Only 1-OH-pyrene was detected, at very low levels, and only in samples from some of the ponds (Fig. 5, Supplementary table S2).

A Pearson correlation analysis revealed no statistically significant relationship between 1-OH-pyrene concentrations and pyrene concentrations (calculated using the sum of concentration in tissue and exuvia) in late instar nymphs (r = − 0.21, p = 0.55, n = 10).

#### Biota-sediment bioaccumulation factors (BSAF)

**Figure 6:**
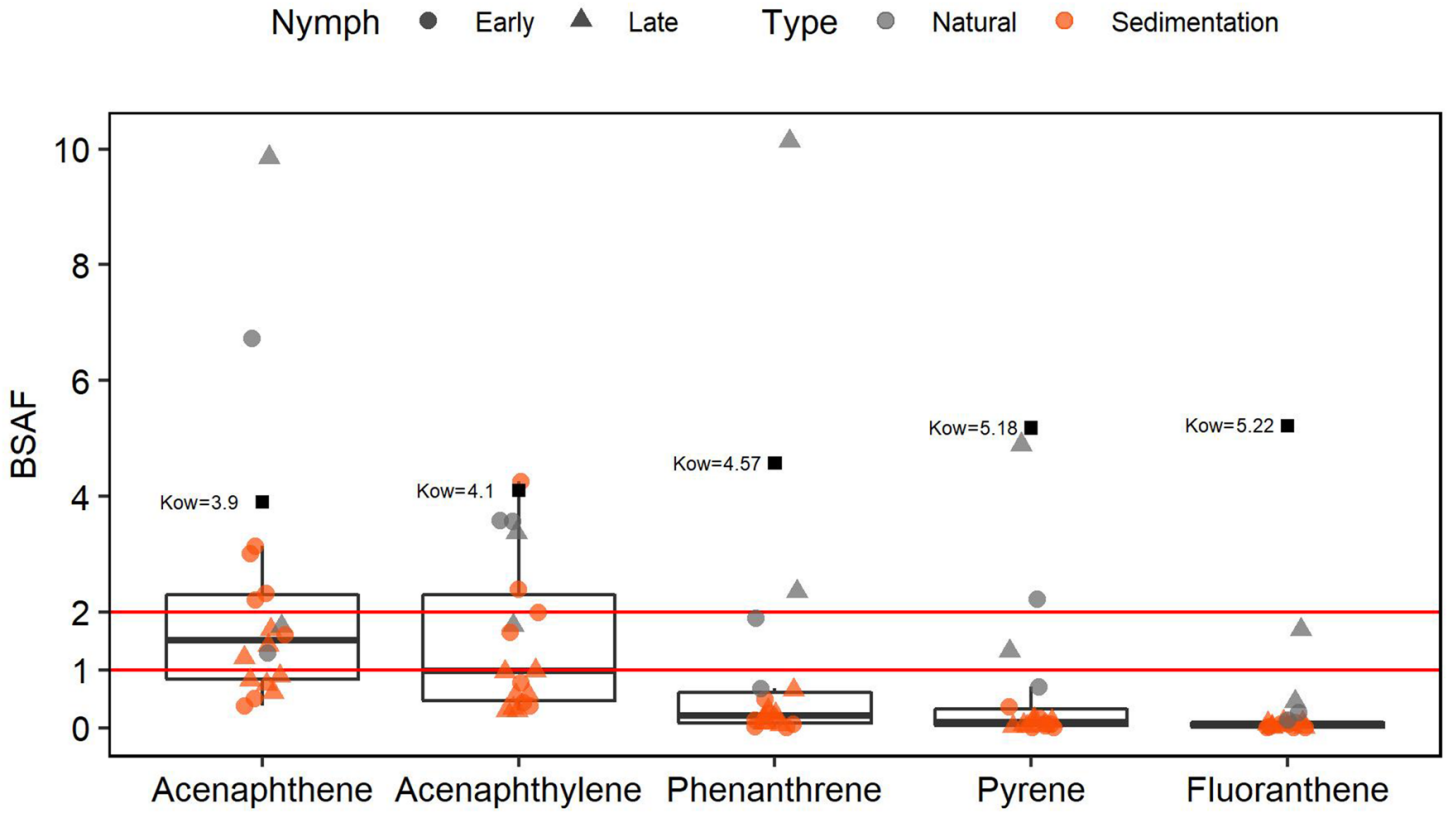
Boxplot of the BSAF. Box represents the 2nd and 3rd quartiles. Black horizontal line represents the median and asterisk represents the mean. Number of points in each plot: Natural: n = 4, Sedimentation; n = 14. Red lines indicate BSAFs = 1 and 2.

**Figure 7:**
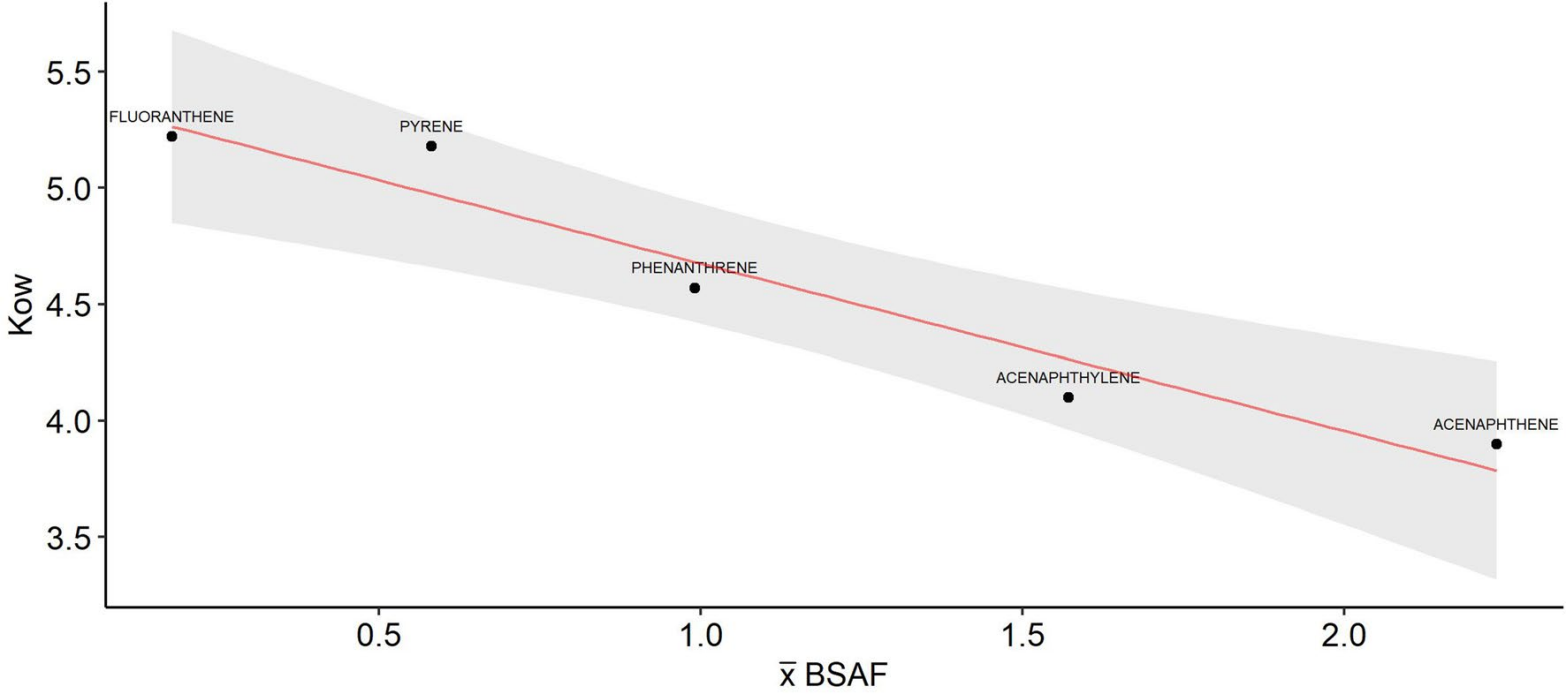
Relationship between the mean BSAF and Kow of PAHs acenaphthene, acenaphthylene, phenanthrene, pyrene, and fluoranthene (n = 5). Fitting line in red. 95% confidence interval represented as grey shading.

BSAF results revealed ratios ranging from approx. 0.006 to 10 (Fig. 6), with a significant difference between PAHs (Kruskal–Wallis, p < 0.001). The highest BSAF values were of acenaphthene and acenaphthylene, followed by phenanthrene. The lowest BSAFs were of pyrene and fluoranthene. Most BSAFs from sedimentation ponds were below 1, whereas in natural urban ponds most BSAFs were above 1. Linear regression analysis showed a strong negative relationship between the mean of BSAFs and K_ow_ (R^2^ = 0.91, p = 0.007; Fig. 7).

## Discussion

### Levels of PAHs and their alkylated homologues in sediment

The results showed a significant difference in concentrations of PAH-16 in sediment, with higher levels being detected in the sedimentation ponds. Consequently, the biota living in these artificial ponds are exposed to higher levels of PAHs than biota living in the ponds that are not directly affected by highway runoff. Previous studies have shown that traffic-related contaminants can lead to negative effects in aquatic organisms inhabiting sedimentation ponds. Examples are fish^3,36^, insects^53^, and amphibians^37^. Therefore, sedimentation ponds may represent a low-quality habitat compared to natural urban ponds. Regression analysis suggested a relationship between the concentrations of PAHs in sedimentation ponds and annual average daily traffic (AADT); R^2^ = 0.61, p = 0.03. The results, however, were highly influenced by SED-7.

Samples from SED—5 and 7 contained the lowest concentrations amongst all sedimentation ponds. PAH-16 concentrations in sediment from SED—7 were exceptionally low. A possible reason for such low concentrations might be that these ponds are not functioning properly, and hence, they may either not be receiving the runoff or not retaining contaminants as predicted. Further examination of these ponds should be considered to investigate their functionality.

In this study we took a step further from the standard analysis of PAH-16 only. The addition of alkylated PAHs to the total sum of PAHs led to a significant increase in measured PAH levels in virtually all sediment samples. The use of a limited list of PAHs as a proxy reduces costs and analytical complexity, which can result in better comparability between analyses performed worldwide^11^. Several studies indicated, however, the effects of alkylated PAHs in aquatic organisms, such as growth inhibition, malformation, reduction of survival rates^38^, and behavioral disruption^39^. A toxicity test performed by Turcotte et al. ^13^ revealed that alkylated forms of phenanthrene were more toxic than its parental form, and that toxicity increased with increase of alkyl substituents. PAHs of petrogenic origin often contain high levels of alkylated PAHs in relation to their parental forms. Therefore, analysis of only parent forms might potentially ignore much higher concentrations of the total PAHs in many situations, distorting the true environmental status, and the potential threat from these contaminants. Based on our results, analysis of a more extensive list of PAHs in environmental samples, as suggested by Andersson & Achten^11^, showed to be useful for road runoff contaminated sites.

### PAHs in dragonfly nymphs

Only parent PAHs and metabolites were analysed in the nymph samples as the separation of exuvia and tissue in late nymphs limited the amount of material available. PAHs were detected in the exuvia, indicating that dragonfly nymphs are able to eliminate PAHs through molting. The results showed, however, a significant difference in the concentration of PAHs in the tissue and exuvia, with over 65% of total PAHs being detected in tissue. Concentrations of PAHs in *Early* and *Late* instars were also significantly different, and higher concentrations were detected in the latter group. Hence, despite the nymph’s capacity to eliminate PAHs through molting, the level of elimination is likely not enough to avoid bioaccumulation.

To the best of our knowledge, this is the first study investigating the biotransformation of PAHs in dragonflies. 1-OH-pyrene was the only metabolite detected, at very low levels, and only in some samples. Since 1-OH-pyrene was the most abundant PAH metabolite found in previous analyses conducted by our research group, it was not a surprise that no other metabolite was detected in the present study. Parent phenanthrene was detected at higher levels in most tissue samples compared the other PAHs found, whilst no metabolites were detected. These results suggest that phenanthrene was not metabolized by the individuals used in this study. It is therefore not clear whether 1-OH-pyrene found in our samples came from external sources or if metabolism of PAHs in dragonfly nymphs is too inefficient to avoid a certain level of bioaccumulation.

The low levels of 1-OH-pyrene detected could also be the result of trophic transfer. Previous studies observed uptake of PAH metabolites through diet^40–43^. Due to invertebrates’ ineffective metabolic processes, metabolites may take longer to be biotransformed from phase I to phase II, thus remaining longer in the prey’s body. In addition, invertebrates excrete compounds slower than vertebrates due to the lack of kidneys^44^. Thus, metabolic PAHs may remain in invertebrate prey long enough to be transferred to predators. Nevertheless, dragonfly nymphs are efficient predators, and vertebrates such as tadpoles are also included in their diet^45^,^46^. Tadpoles were spotted in almost all ponds included in this study. Macroinvertebrates biotransform PAHs at different efficiency levels^24–26,47^. A better understanding of the diet of the nymphs in these ponds could perhaps explain why 1-OH-pyrene were detected in some samples, but not in others. The potential trophic transfer is of concern due to the toxic nature of PAH metabolites.

Despite our results suggesting bioaccumulation of PAHs in dragonfly nymphs, it is possible that these contaminants are not transferred from the nymph/aquatic to the adult/terrestrial stage. Wayland et al. ^48^ observed similar levels of PAHs in damselfly adults and nymphs (Odonata: Zygoptera). Lower levels would be expected in the nymphs if bioaccumulation was a factor. The study suggests that bioaccumulation of PAHs in adult damselflies’ might be mostly due to the insect’s feeding habits, and less from possible bioaccumulation during their aquatic life stage. In addition, a study from Kraus et al. ^49^ on biomagnification of contaminants in insects demonstrated a great loss of PAHs during metamorphosis, with nymph concentrations being up to 125-fold higher than in adults. As hemimetabolous species, dragonflies go through a major transition during metamorphosis. Their aquatic and terrestrial life stages are associated with completely different lifestyle and appearance. While their nymph stage specializes in growth, their adult life is specialized for dispersal and reproduction^50^. It is, therefore, reasonable to suggest that bioaccumulation might occur only during the dragonfly’s nymph stage.

#### Biota-sediment bioaccumulation factors (BSAF)

This study observed BSAF values as low as 0.006 and as high as 10. Our results may, however, be subject to uncertainty since we were unable to determine lipid content for nymphs from individual sites due to limited material available. Nevertheless, as our samples are most likely of the same species, and we grouped the individuals according to similar size and weight, we do not expect much variation in lipid content based on site location. In addition, previous analysis performed by our team in nymphs from individual sites (including some sites used in this study) showed a lipid variation of only 1.6% (unpublished results).

Our results showed that BSAFs decreased with increase of PAH’s K_ow_, in line with previous studies^51,52^, and the correlation between BSAF and partition coefficient has been suggested as a BSAF predictor for bottom dwellers^53^. Virtually all 5- and 6-ring PAHs in dragonfly nymphs were below quantification levels. This was not a surprise, as heavier PAHs tend to strongly adsorb to sediment^54,55^. The only 4-ring PAHs quantified were pyrene and fluoranthene, which have K_ow_ values below 5.5. Thus, our results suggest that less hydrophobic PAHs (i.e. those with relatively low K_ow_) might not adsorb strongly enough to sediment at certain conditions, being as a result more bioavailable. Low molecular weight PAHs might be dissolved in water or adhered to suspended particles (< 62 µm). Since surface area is inversely proportional to particle size, smaller particles have a higher contaminant-carrying capacity than larger particles^56,57^. In an experiment in which the distribution of PAHs from road runoff was quantified in particulate fractions, Nielsen et al. ^58^ detected a significant amount of low- and middle molecular weight PAHs in the colloidal and dissolved fractions in traffic runoff.

BSAF for PAHs are often reported in bottom-dwelling filter feeders. Nonetheless, *Aeshna* species might be highly exposed to sediment as they live very close to the bottom, and most likely include bottom-dwelling organisms in their diet. In addition, their gills are placed inside their rectal chamber; thus water, and unavoidably sediment particles, are pumped inside their bodies during respiration^59^.

Pyrene, fluoranthene, and phenanthrene were accounted for the most part of the observed toxicity in freshwater amphipods exposed to highway runoff in tests that also included anthracene, chrysene, and benzo[a]anthracene^60^. Carls et al. ^61^ demonstrated the toxic effects of dissolved petrogenic PAHs by exposing fish embryos indirectly to oil droplets that were kept isolated by using an agarose barrier. Thus, analysis of PAHs in water and suspended particles should be considered in future studies to investigate the bioavailability of these organic compounds.

The BSAF/K_ow_ relationship might, however, be due to other factors other than the adsorption capacity of PAHs. For instance, Thomann and Komlos^52^ observed that low BSAFs for PAHs with K_ow_ > 5 in sunfish (also a predator) were primarily due to low gut assimilation capacity and high metabolism efficiency.

BSAF values were generally higher in natural urban ponds compared to sedimentation ponds. This could be the result of the selected PAHs being more bioavailable in natural urban ponds. Previous studies have shown that BSAFs may be influenced by physical–chemical characteristics of the sediment^62^, ecological characteristics^63^, and the contaminant’s K_ow_^64^. BSAFs might therefore be a useful tool to indicate the contaminant-retaining functionality of the sedimentation ponds. To support this contention, the sedimentation ponds with the highest overall BSAF ratios were the same as those with particularly low PAH concentration detected in sediments (SED—5 and 7). Nevertheless, Meland et al. ^65^ observed higher levels of DNA damage in dragonfly nymphs (*Aeshna *sp.) living in sedimentation ponds compared to natural urban ponds. In addition, a strong correlation between levels of DNA damage and PAH levels in sediment was observed in the same study.

PAHs were detected in significant higher levels in dragonfly nymph tissues than in exuvia, indicating some level of bioaccumulation of PAHs in dragonfly nymphs. In addition, it is not clear whether dragonfly nymphs biotransform PAHs inefficiently, or if the 1-OH-pyrene detected was a product of trophic transfer. Further studies would be needed to answer this question.

Our results showed that the inclusion of alkylated PAHs drastically increased the overall measured PAH levels in sediments in all ponds, and thus gave a more realistic picture of the status of the ponds studied. Previous studies have observed that BSAF for alkylated PAHs are often > 1^66^, and in some cases they were greater than for parent PAHs with similar K_ow_^52^, including for dragonfly nymphs^48^. In this study we determined BSAF for parent PAHs only. As our results suggest, alkylated PAHs comprise a large percentage of the overall PAH in sediments contaminated by road runoff. It is therefore important to include alkylated homologues when determining BSAF from species exposed to such sediments.

Sedimentation ponds are oases for research on the effects and fate of contaminants in the environment. It is crucial, however, that their ecological role is considered during the design and construction process to enhance their capacity as suitable habitats, and ensure that they remain an environmental solution instead of an environmental burden.

## Materials and methods

### Study sites

**Figure 8:**
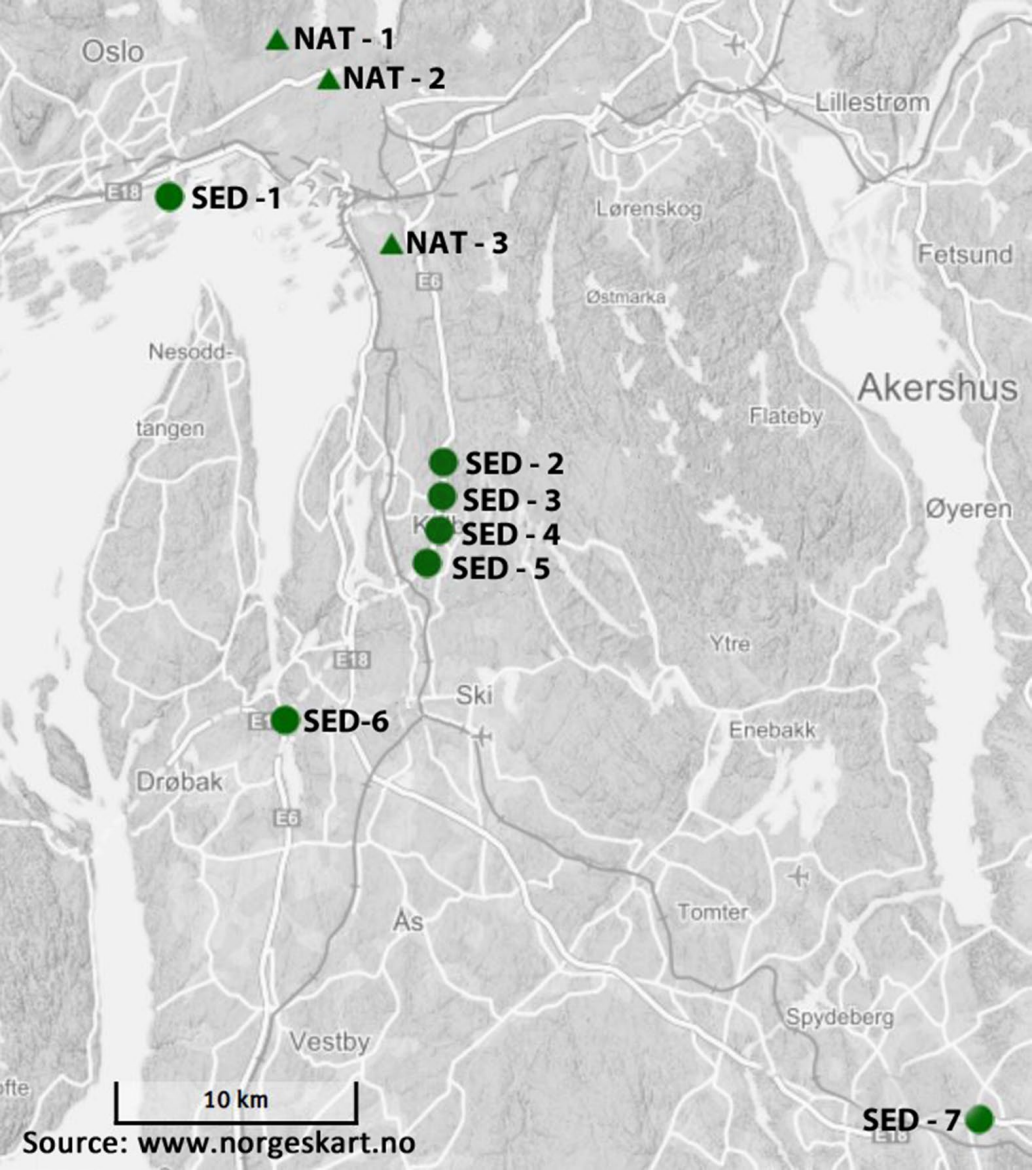
Map showing the location of the ponds. Sedimentation ponds are marked with circles and natural urban ponds are marked with triangles. Map was obtained from the Norwegian Mapping Authority’s online map service (www.norgeskart.no).

Nymphs and sediment samples were collected from seven sedimentation ponds and three natural urban ponds situated in the counties of Oslo, Akershus, and Østfold, in Norway (Fig. 8). Sedimentation ponds were labelled as SED—1 to 7, and natural ponds as NAT -1 to 3 (Table 2).

### Sampling

Samples were collected in June 2018, except for the early instars and sediment from NAT—3, which were collected in June 2017. All samples were transported to the laboratory in glass jars sterilized at 550 °C for 30 min. Sediment samples were collected from approximately the top 5 cm of each location with a van Veen grab. Each sediment sample consisted of material collected from five different spots which were combined and mixed. Nymphs were transported in polystyrene boxes with ice. Once at the laboratory, nymphs were rinsed with distilled water, pat dried, and killed by introducing a scalpel to the head. They were then weighed and measured (Table 3). Nymphs were identified to be from the Genus *Aeshna,* as described by Brooks & Cham^67^. Individuals were divided into groups *Early instars* and *Late instars* (supplementary figure S1).* Late instars* were inferred to be individuals in instars F1–F0 (penultimate and final instars, respectively), and *Early instars* were those in earlier developmental stages. Stages were determined by using the minimum and maximum size values for body and wing pad length for *Aeshna cyanea* as determined by Goretti et al. ^68^. Samples were stored in sterilized jars (550 °C for 30 min), except for haemolymph, which was kept in glass capillaries. Sampled materials were pooled in order to obtain enough material to detect contaminants (Table 3), and kept at − 20 °C until further analysis.

**Table 3:** Number of nymphs pooled from each pond. In addition, mean weight and length are displayed.

**Ponds**	**Late instar nymphs**			**Early instar nymphs**		
	**Nr**	**Weight (g) (mean ± SD)**	**Length (cm) (mean ± SD)**	**Nr**	**Weight (g) (mean ± SD)**	**Length (cm) (mean ± SD)**
SED—1	15	0.97 ± 0.10	3.89 ± 0.27	3	0.34 ± 0.11	2.68 ± 0.55
SED—2	12	1.11 ± 0.12	4.13 ± 0.21	6	0.59 ± 0.06	3.05 ± 0.26
SED—3	6	1.01 ± 0.13	3.92 ± 0.24	11	0.46 ± 0.17	3.19 ± 0.43
SED—4	15	1.06 ± 0.09	3.96 ± 0.14	2	0.47 ± 0.18	3.00 ± 0.42
SED—5	17	1.05 ± 0.09	4.01 ± 0.23	10	0.21 ± 0.11	2.27 ± 0.18
SED—6	12	0.93 ± 0.11	3.95 ± 0.18	7	0.25 ± 0.07	2.51 ± 0.31
SED—7	10	0.98 ± 0.12	3.99 ± 0.16	9	0.29 ± 0.17	2.42 ± 0.54
NAT—1	10	1.06 ± 0.31	4.08 ± 0.46	13	0.40 ± 0.13	3.03 ± 0.38
NAT—2	11	1.07 ± 0.16	3.91 ± 0.18	0	–	–
NAT—3	9	1.04 ± 0.17	3.87 ± 0.28	4	0.51 ± 0.15	3.2 ± 0.3
Sum nymphs in sedimentation ponds	87	1.02 ± 0.12	3.98 ± 0.22	48	0.35 ± 0.18	2.5 ± 0.4
Sum nymphs in natural urban ponds	30	1.06 ± 0.22	3.96 ± 0.33	17	0.43 ± 0.13	3.2 ± 0.3
Total no. of nymphs	117	1.03 ± 0.15	3.98 ± 0.25	65	0.37 ± 0.11	2.6 ± 0.2

#### PAH determination

##### Extraction and clean-up

PAHs were analysed in all samples. Due to limited biota material, alkylated homologues were analysed only in sediment. Nymphs in the *Late instars* group had exuvia and tissue analysed separately, and haemolymph extracted for determination of PAH metabolites. *Early instars* were analysed as a whole. Exuvia and the internal tissues were separated in frozen individuals. Internal tissues were scraped out with a metal spoon. Wing pads were pulled off to get access to the wings, which were already developed in some individuals. Tissue and exuvia were transferred to separately marked extraction glasses for further analysis.

Approximately 5 g of sediment and 2–8 g of nymphs (split between tissues and exuvia if late instars) were freeze-dried for 48 h. Dried contents were homogenized with a glass stirring rod and mixed with approximately 15 mL of cyclohexane: dichloromethane (90:10). 50 µL of PAH internal standard (2 µg/mL, dissolved in toluene) were added. The internal standards were naphthalene-d8, biphenyl-d10, acenaphthylene-d8, dibenzothiophene-d10, pyrene-d10, benzo(a)anthracene-d12, and perylene-d12. The following procedure was done twice, and extracts combined: The extracts were placed in an ultrasonic bath for 1 h and centrifuged for 5 min at 3,000 RPM. Extracts were concentrated to 1 mL in an automated solvent evaporation system (TurboVap LV) at 37 °C, transferred to 2 mL vials, and further concentrated to approx. 100 µL. Next, 400 µL of ethyl acetate (LS-MS graded) was added. Extracts were transferred to polypropylene microcentrifuge tubes with centrifuge tubes filters (0.2 µM nylon filters), centrifuged for 1 min at 13,000 RPM, and transferred to vials. A small amount of cyclohexane was added, and the extracts were further concentrated by a gentle flow of nitrogen to approximately 100 µL before being transferred to 0.9 mL vials for analysis.

Biota extract was cleaned by Gel Permeation Chromatography (Agilent Technologies, Wilmington, DE, USA), with a 300 × 7.5 mm column of the type PLgel 10 um, 100 Å (pore size). Fraction collected was between 4.8–11.3 µL/min at 50 °C. Mobile phase consisting of ethyl acetate: cyclohexane 80:20, and flow of 2 mL/min was used. Sediment and biota extracts were analysed by gas chromatography/mass spectrometry operated in single ion monitoring mode (SIM—Agilent GC 6,890/MSD 5,973; Agilent Technologies, Wilmington, DE, USA). The internal standard method was used for quantification of individual components. LODs were determined by the “signal to noise” method as described by Shrivastava and Gupta^69^.

##### GC/MS analysis

The analyses were performed as described in Meland et al. ^65^. In brief, we used gas chromatography/mass spectrometry (Agilent GC 6890/MSD 5973; Agilent Technologies, Wilmington, DE, USA) operated in single ion monitoring mode (SIM). The ionization was electron impact (70 eV). PAHs were individually separated on a DB5 column (30 m, 0.25 mm inner diameter, and 0.25 µm film thickness: Agilent JW Scientific). The injection was pulsed splitless injection (2 µL injection, pulse pressure 20 psi for 1.2 min, injection temperature 300 °C), and the carrier gas was He (1.2 mL/min). The temperature of the GC oven started at 60 °C for the first 2 min, then further raised to 250 °C (7 °C/min), and was finally raised to 310 °C (15 °C/min), being kept at that temperature for 6 min. Temperatures for the quadrupole, ion source, and transfer line were 150 °C, 230 °C, and 300 °C respectively. Concentrations of alkylated PAHs were estimated using the response factors of the corresponding parent PAH. Limit of detection (LOD) ranged from 2 to 1,100 µg/kg for sediment, and 1 to 30 ng/g for biota.

#### PAH metabolites

##### Extraction and clean-up

Haemolymph was extracted by removing the middle leg of the nymphs and applying gentle pressure for its release into a glass capillary (Hilgenberg, 80 mm length, 0.4 mm outer circumference, 0.04 mm wall thickness). The volume of haemolymph extracted was measured by its weight. Approximately 10 µL of haemolymph were transferred to polypropylene microcentrifuge tubes, followed by 10 µL (1,000 µg/mL) of internal standard; triphenylamine. During the procedure, the samples were kept on ice whenever possible, whilst kept away from direct light. 50 µL of Milli-Q water (18.2 MΩ·cm resistivity with Millipak membrane filter 0.22 µm; Merck KGaA, Germany) was added to the samples, followed by 20 µL of β-glucuronidase (200 U/µL haemolymph) with aryl sulfatase activity (10 U/µL haemolymph). Samples were set on a heating block at 37 °C for 1 h, and then 200 µL of methanol was added and mixed well. Samples were centrifuged for 10 min at 13,000 RPM, and supernatants were transferred to 300 µL vials and kept in a freezer at − 20 °C until analysis.

##### HPLC analysis

Metabolite analysis was performed as described in Grung et al. ^3,36^. Samples containing 25 µL of haemolymph extract were analysed by high performance liquid chromatography (HPLC) using Waters 2,695 Separations Module with a Waters PAH C18 (4.6 × 250 mm, 5 µm) column and 2,475 fluorescence detector. Calibration standards from Chiron AS, Trondheim, Norway (0.2–200 ng/g. range), and internal standard method were used for quantification of individual components. Mobile phase was comprised of a gradient starting from 40:60 acetonitrile : ammonium acetate aqueous solution (0.05 M, pH 4.1) to 100% acetonitrile gradient at a flow of 1 mL/min (total time of 60 min), and column temperature of 35 °C. Fluorescence excitation/emissions: 1-OH-phenanthrene 256/380; 1-OH-pyrene 346/384; triphenylamine 300/360; 3-OH-benzo[a]pyrene 380/430). LOD ranged between 0.1 and 3 ng/g (0.18 ng/g bile for 1-OH-pyrene, which is the PAH metabolite most often detected). This is quite low in comparison with many analyses of PAH-metabolites performed by us in bile from fish, where the LOD were up to 5 times this level^36^. Fish bile, however, generally contains a significant number of compounds that might cause chromatographic noise. There was less background noise in the haemolymph chromatograms compared to bile, thereby facilitating a lower LOD than usual in our lab. Since PAHs are detected in the nymphs, we were expecting low or non-detectable levels of PAH-metabolites. We have also previously analysed blue mussels (*Mytilus edulis;* unpublished results), and, as in the case of dragonfly nymphs used in this study, PAH-metabolites were detected at very low levels. Blue mussels are species known for their accumulation of PAHs and are used as sentinel organisms in monitoring of oil pollution.

### Biota-sediment accumulation factor (BSAF)

BSAF is the ratio of the concentration of a contaminant in the organism and sediment. BSAF for each PAH was measured in early and late instar samples using the following equation:

$$\frac{\left(\frac{{c}_{biota}}{{f}_{lip}}\right)}{\left(\frac{{c}_{sed}}{{f}_{oc}}\right)}$$

where C_biota_ is the concentration of PAH in the biota (early and late instars, mg/kg, dry weight), *f*_lip_ is the fraction of lipid content in the biota (mg/kg, dry weight), C_sed_ is the concentration of PAH in the sediment (mg/kg, dry weight), and *f*_oc_ is the fraction of organic content in the sediment (mg/kg, dry weight). *f*_lip_ and *f*_oc_ were analysed by the Department of Organic Chemistry at NIVA. *f*_lip_ was determined by pooling same-size nymphs from all ponds (one fraction for early and one for late instars). BSAF for NAT2 was not calculated because we did not have *f*_oc_ for this sediment sample.

For benthic organisms, partition equilibrium of a chemical between sediment and organism is expected at BSAF values between 1 and 2^70^.

### Quality assurance

Quality assurance was performed as described by Meland et al. ^65^. In short, we analysed two samples of Standard Reference Material (SRM) and three blank samples (containing internal standards) with every batch in order to trace potential sample contamination or loss of contaminants. For sediment analysis we used NIST SRM 1944 with an average deviation of − 11% (min. − 32%, max. 9%). For PAH analysis in nymphs NIST SRM 2974a was used, with average deviation of − 12.7% (min. − 2.8%, max. − 20.3%). For metabolite analysis we used reference material prepared at NIVA, and the average deviation was of − 4.5% (min. − 62%, max. 25%).

### Statistical analysis and data handling

Statistical analyses were performed using RStudio (version 1.1.456-2009-2018) and JMP software (SAS Institute, version 14.0.0-2018).

For dragonfly data, only PAHs which had at least 80% of the samples quantified were used. Observations reported as “less than” ( <) were substituted with half of its value. For sediment and haemolymph data, different substitution methods (as described in Wood, Beresford & Copplestone^71^) were tested, with no significant difference in the overall results. Consequently, [( <)/2] was also applied for variables containing less than 80% of observations detected, when appropriate.

Significance level was set to α = 0.05 for all statistical tests. Shapiro–Wilk tests for normal distribution were performed. Data were log-transformed when normality assumptions were not met. Welch's t-test was performed whenever the normality assumption was met. The Spearman's rank-order was used for correlation analysis.

## Supplementary information

Supplementary file1 (DOCX 698 kb)

## Data Availability

Datasets are available from the authors upon request.
