# Supplementary material for: Polycyclic aromatic hydrocarbons: bioaccumulation in dragonfly nymphs (Anisoptera), and determination of alkylated forms in sediment for an improved environmental assessment
Source: Sci Rep. 2020 Jul 2;10:10958. doi: 10.1038/s41598-020-67355-1 (PMC7331706; doi:10.1038/s41598-020-67355-1)
Supplement: Supplementary file 1 — Supplementary file1 (DOCX 698 kb) [file 41598_2020_67355_MOESM1_ESM.docx]

# Supplementary Information

**Polycyclic aromatic hydrocarbons: bioaccumulation in dragonfly nymphs (Anisoptera), and determination of alkylated forms in sediment for an improved environmental assessment**

**Viviane Girardin^1^*, Merete Grung^1^, Sondre Meland^,1,2^**

^1^Norwegian Institute for Water Research (NIVA), Gaustadalléen 21, 0349 Oslo, Norway

^2^Norwegian University for Life Sciences (NMBU), Faculty of Environmental Sciences and Natural Resource Management, PO 5003, 1432 Ås, Norway

*Corresponding author: Viviane Girardin, Norwegian Institute for Water Research (NIVA), Gaustadalléen 21, 0349 Oslo, Norway. E-mail address [viviane.girardin@niva.no](mailto:viviane.girardin@niva.no), phone +47 970 37 586


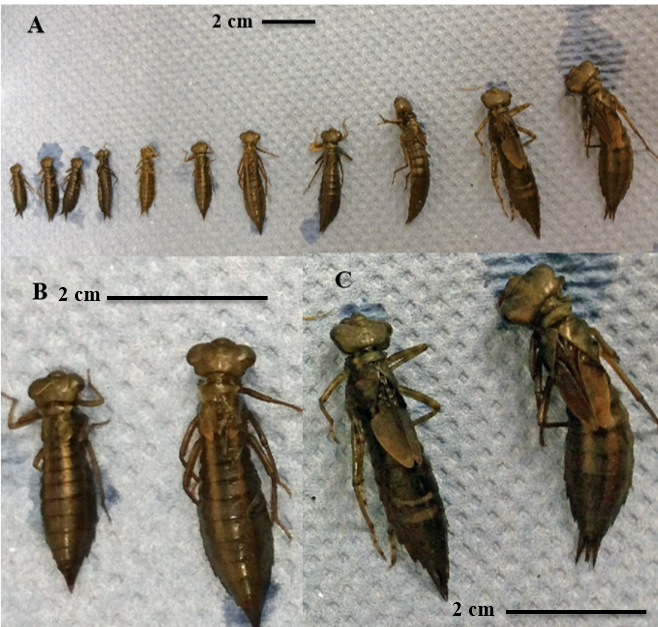


Supplementary figure S1 - Larvae were divided into two groups according to their length and wing pad length. A –Larvae ranging from approximately 2 cm to 4.5 cm. B – Examples of larvae from group Early instars. C – Examples of larvae from group Late instars.

Supplementary table S1- PAH levels in dragonfly nymphs (ng/g, dry weight). Small nymphs were not found in NAT-2.

|  | PAHs | SED - 1 | SED - 2 | SED - 3 | SED - 4 | SED - 5 | SED - 6 | SED - 7 | NAT - 1 | NAT - 2 | NAT - 3 |
| --- | --- | --- | --- | --- | --- | --- | --- | --- | --- | --- | --- |
| Tissue | Acenaphthylene | 34 | 22.3 | 13.8 | 30.5 | 29.1 | 40.1 | 17.7 | 22.7 | 14.7 | 18.1 |
|  | Acenaphthene | 15 | 15 | 7.8 | 17 | 19 | 34 | 7 | 13 | 12 | 8.3 |
|  | Phenanthrene | 31 | 36 | 15 | 43 | 24 | 45 | 79 | 57 | 20 | 26 |
|  | Fluoranthene | 13 | 11 | 4.6 | 10 | 6.4 | 16 | 48 | 35 | 5.3 | 6.3 |
|  | Pyrene | 46 | 33 | 24 | 17 | 13 | 34 | 123 | 49 | 18 | 12 |
| Exuvia | Acenaphthylene | 20 | 8.3 | 4.7 | 22.1 | 12.2 | 8.1 | 5.8 | 7.5 | 7.5 | 6.3 |
|  | Acenaphthene | 11.9 | 4.8 | 6.8 | 19 | 12 | 5.7 | 4.2 | 5.5 | 5.7 | 3.5 |
|  | phenanthrene | 15 | 12 | 11 | 21 | 17 | 17 | 27 | 18 | 11 | 13 |
|  | Fluoranthene | 4.1 | 3.5 | 3.2 | 6.5 | 4.3 | 8.8 | 20 | 15 | 2.2 | 4.9 |
|  | Pyrene | 7.4 | 31 | 4.7 | 19 | 9.2 | 15 | 47 | 8.6 | 18 | 13 |
| Whole | Acenaphthylene | 25.7 | 16.8 | 43.3 | 12.4 | 13 | 44.5 | 34.1 | 8.2 | - | 9.6 |
|  | Acenaphthene | 24 | 12 | 47 | 10 | 23 | 30 | 64 | 7.7 | - | 4.2 |
|  | phenanthrene | 26 | 17 | 30 | 10 | 21 | 17 | 43 | 15 | - | 5 |
|  | Fluoranthene | 14 | 5.2 | 7.8 | 3.6 | 4.6 | 9.7 | 32 | 11 | - | 1.2 |
|  | Pyrene | 53 | 8.6 | 57 | 9 | 37 | 25 | 75 | 21 | - | 7.8 |

Supplementary table S2- 1-OH-pyrene levels in dragonfly nymphs (ng/g, wet weight). LOD = 0.18

| **Pond** | **1-OH-pyrene** |
| --- | --- |
| SED - 1 | 0.376 |
| SED - 1 | 0.971 |
| SED - 1 | 0.83 |
| SED - 1 | 0.69 |
| SED - 2 | 0.106 |
| SED - 2 | 0.094 |
| SED - 3 | 0.147 |
| SED - 4 | 0.843 |
| SED - 5 | 0.657 |
| SED - 5 | 0.654 |
| SED - 5 | 0.55 |
| SED - 6 | 1.837 |
| SED - 6 | 1.2 |
| SED - 6 | 1.7 |
| SED - 7 | 0.047 |
| SED - 7 | 0.038 |
| SED - 7 | 0.049 |
| NAT - 1 | 0.873 |
| NAT - 1 | 0.41 |
| NAT - 2 | 0.151 |
| NAT - 2 | 0.049 |
| NAT - 3 | 0.062 |
